# Making the final cut: pathogenic amyloid-β peptide generation by γ-secretase

**DOI:** 10.15698/cst2018.11.162

**Published:** 2018-10-28

**Authors:** Harald Steiner, Akio Fukumori, Shinji Tagami, Masayasu Okochi

**Affiliations:** 1 Biomedical Center (BMC), Metabolic Biochemistry, Ludwig-Maximilians-University Munich, Germany.; 2 German Center for Neurodegenerative Diseases (DZNE), Munich, Germany.; 3 Department of Aging Neurobiology, National Center for Geriatrics and Gerontology, Obu & Department of Mental Health Promotion, Osaka University Graduate School of Medicine, Toyonaka, Japan.; 4 Neuropsychiatry, Department of Integrated Medicine, Division of Internal Medicine, Osaka University Graduate School of Medicine, Suita, Japan.

**Keywords:** Alzheimer's disease, amyloid β-peptide, presenilin, γ-secretase

## Abstract

Alzheimer´s disease (AD) is a devastating neurodegenerative disease of the elderly population. Genetic evidence strongly suggests that aberrant generation and/or clearance of the neurotoxic amyloid-β peptide (Aβ) is triggering the disease. Aβ is generated from the amyloid precursor protein (APP) by the sequential cleavages of β- and γ-secretase. The latter cleavage by γ-secretase, a unique and fascinating four-component protease complex, occurs in the APP transmembrane domain thereby releasing Aβ species of 37-43 amino acids in length including the longer, highly pathogenic peptides Aβ42 and Aβ43. The lack of a precise understanding of Aβ generation as well as of the functions of other γ-secretase substrates has been one factor underlying the disappointing failure of γ-secretase inhibitors in clinical trials, but on the other side also been a major driving force for structural and in depth mechanistic studies on this key AD drug target in the past few years. Here we review recent breakthroughs in our understanding of how the γ-secretase complex recognizes substrates, of how it binds and processes β-secretase cleaved APP into different Aβ species, as well as the progress made on a question of outstanding interest, namely how clinical AD mutations in the catalytic subunit presenilin and the γ-secretase cleavage region of APP lead to relative increases of Aβ42/43. Finally, we discuss how the knowledge emerging from these studies could be used to therapeutically target this enzyme in a safe way.

## INTRODUCTION

Alzheimer´s disease (AD) is the most common form of dementia worldwide currently affecting about 47 millions people. The disease affects the elderly typically above an age of ~65 and manifests with progressive memory impairment and cognitive decline. About a third of the people at an age of 85 are diagnosed with AD. Aging is thus the most important risk factor for the disease. In very rare cases, AD can also be genetically inherited. These familial forms of AD (FAD) are typically characterized by a much earlier onset of the disease below 65 years, which can occur in very aggressive forms already in adolescence. To date, there is no therapy available that can prevent the disease or cure it 1. 

**Figure 1:**
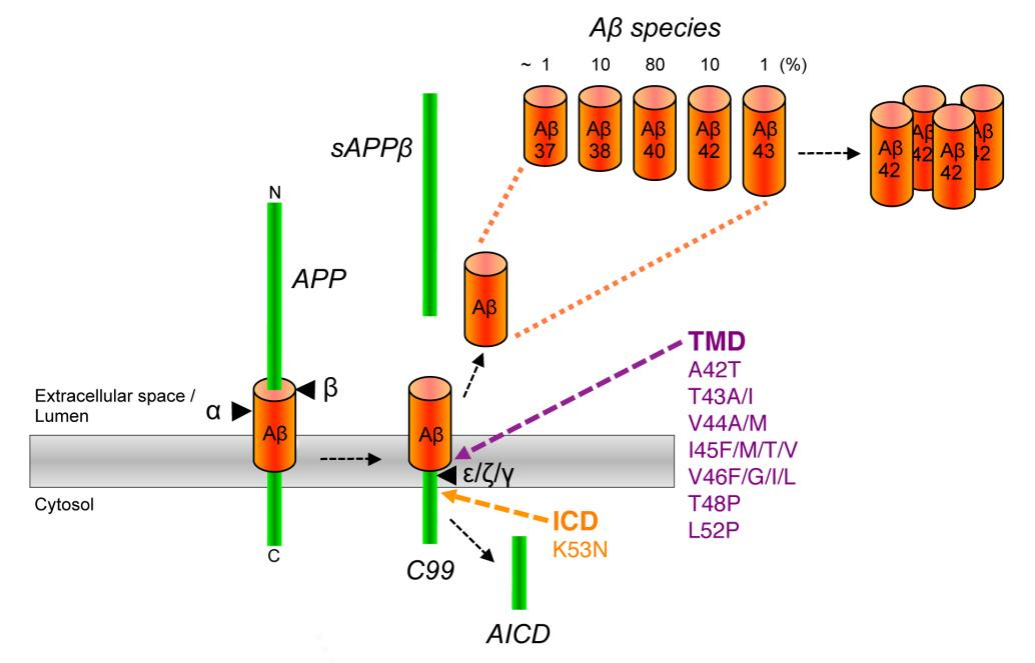
FIGURE 1: APP processing and generation of Aβ. APP is first cleaved by β-secretase (β) in its ectodomain close to the extracellular/luminal membrane border thereby generating a 99 amino acid C-terminal APP fragment (C99). Consecutive cleavages of C99 by γ-secretase at ε-, ζ-, and γ-sites releases the APP intracellular domain (AICD) into the cytosol and 37-43 amino acid Aβ species into the extracellular space or lumen of secretory pathway organelles. Longer Aβ forms such as in particular Aβ42 are highly aggregation prone and ultimately deposit as plaques in AD patient brains. An alternative cleavage of APP by α-secretase (α) in the Aβ domain prevents the formation of Aβ. Pathogenic APP FAD mutations that have been shown or are likely to cause relative increases in the generation of Aβ42 species are located in the γ-secretase cleavage region of the APP TMD and in the AICD.

Pathologically, AD is characterized by a massive deposition of abnormal protein aggregates present as extracellular plaques of amyloid-β peptide (Aβ) in the brain parenchyma and as neurofibrillary tangles, intraneuronal deposits composed of the microtubule binding protein tau 2. In addition, dystrophic neurites and neuropil threads of tau are commonly observed in AD. It is widely believed that abnormal levels of Aβ trigger the disease 3. Aβ is a small hydrophobic ~4 kDa peptide, which is derived by sequential proteolytic processing of the amyloid precursor protein (APP), a single pass transmembrane protein with type I topology which is expressed in neurons as 695 amino acid splice variant 4 (**Fig. 1**). In the first step, APP is cleaved by β-secretase. This cleavage, which is carried out by the membrane-bound aspartyl protease BACE (β-site APP-cleaving enzyme) 5, removes the bulk of the APP ectodomain and leaves a 99 amino acid C-terminal fragment (CTF) in the membrane. This C99 or APP CTFβ termed fragment is cleaved in the next step by a membrane protein complex termed γ-secretase 6. This intramembrane-cleaving aspartyl protease complex cleaves C99 in the transmembrane domain (TMD) at the ε-site thereby releasing the APP intracellular domain (AICD) from the membrane into the cytosol. In a stepwise process, which will be explained in detail further below, a variety of Aβ forms are generated by additional γ-secretase cleavages at the ζ- and γ-sites that trim the TMD 7 (**Fig. 1**). Trimming occurs until the TMD is short enough to release predominantly 38-42 amino acid long Aβ forms from the membrane into the extracellular space or into the lumen of secretory pathway organelles. Aβ40 is generated as major product along minor amounts of the shorter Aβ38 and the longer Aβ42. Besides these species, very little amounts of Aβ37 and Aβ43 are generated. Generation of Aβ is prevented by an alternatively occurring APP cleavage by α-secretase 8, which cleaves within the Aβ region and generates the shorter APP CTF, C83. 

Although Aβ42 is normally generated only in minor amounts, it is the initially and major Aβ form deposited in plaques 9. It is more hydrophobic than Aβ40 and displays a considerably higher propensity to form neurotoxic aggregates and is thus believed to be the culprit of AD pathogenesis 2. A central pathological role of Aβ42 is evident from the finding that mutations associated with FAD were identified in APP and presenilin 10,11,12, the catalytic subunit of γ-secretase 6,13. They affect the cleavage of γ-secretase such that increased amounts of the pathogenic Aβ42 relative to Aβ40 are generated 14,15,16. Missense mutations in APP were the first reported FAD mutations 17,18. Most of these mutations are found within the APP TMD in the γ-secretase cleavage region and like presenilin FAD mutations increase the Aβ42/Aβ40 ratio 19 (**Fig. 1**). Mutations were also found that increase the total amounts of Aβ generated. A double mutation at the β-secretase cleavage site occurring in a Swedish family 20,21 and more recently, also a duplication of the APP gene could be linked to FAD 22. Interestingly, another mutation close to the β-secretase cleavage site that lowers Aβ levels and protects against AD has been identified in the Icelandic population 23. FAD mutations were also found in the N-terminal and mid-domain of Aβ 19. These mutations increase the aggregation properties of Aβ. Also for the more common sporadic forms of AD, genetic components, which increase the risk of developing the disease, have been identified. These include the ε4 allele of the apolipoprotein E (APOE) gene, which affects Aβ aggregation and/or clearance 24 as well as the microglial protein TREM2 (triggering receptor expressed on myeloid cells 2) that is involved in the clearance of Aβ plaques 25. Finally, a central role of Aβ in the etiology of AD is further supported by genome-wide association studies of AD, which indicate a role of genes involved in lipid metabolism and intracellular protein trafficking - all factors, which affect Aβ metabolism 26. 

Since γ-secretase makes the final cut in the generation of Aβ, it is obvious that the enzyme is one of the major drug targets in AD. We will now have a closer look at its structure and functions and in particular at the mechanism of how it generates pathogenic, longer Aβ species in the following chapters.

## STRUCTURE AND FUNCTION OF γ-SECRETASE IN AD AND BEYOND 

**Figure 2:**
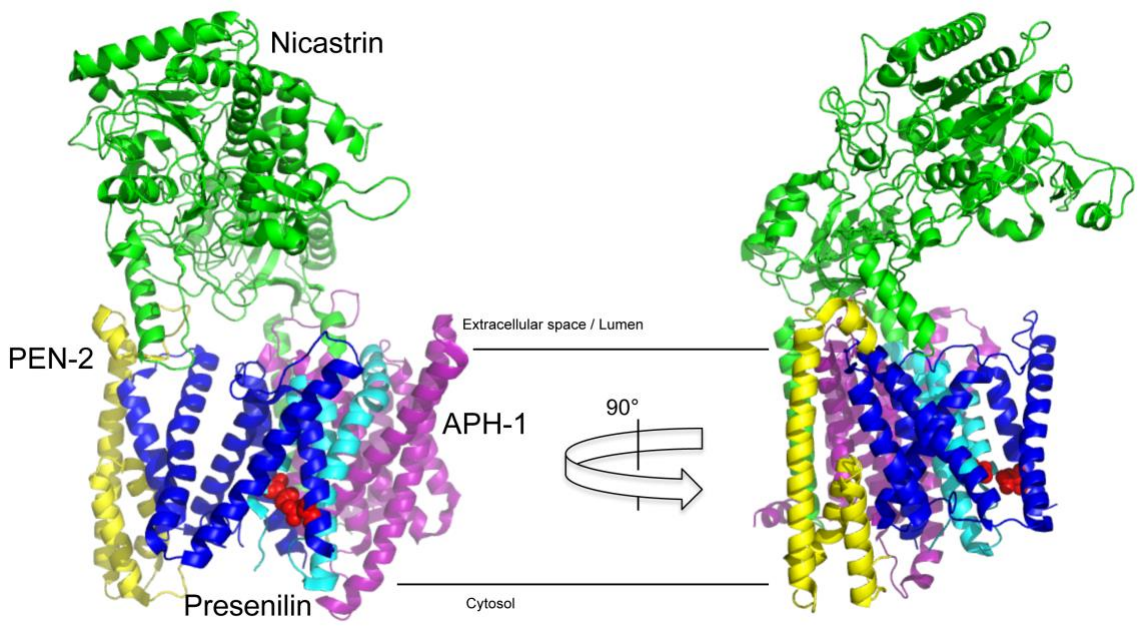
FIGURE 2: Structure of γ-secretase. The atomic resolution structure of γ-secretase (PDB: 5FN3) shows a membrane embedded core containing the catalytic subunit presenilin-1 cleaved into NTF (blue) and CTF (cyan) flanked by the subunits PEN-2 (yellow) and APH-1a (purple), which is covered by the large bilobar extracellular domain of the nicastrin subunit (green). This domain allows nicastrin to serve as gatekeeper controlling substrate access to the active site by excluding proteins with too large (or sterically incompatible) ectodomains. Red spheres depict the active sites aspartate residues.

γ-Secretase is a protein complex composed of four subunits 27,28,29, whose structure has recently been solved at atomic resolution by cryo-electron microscopy 30 (**Fig. 2**). The largest subunit nicastrin (NCT) is a type I membrane protein 31, whose large bilobar highly glycosylated extracellular domain covers the horseshoe-like transmembrane part of the complex 32. The catalytic subunit of the complex, presenilin, is a polytopic membrane protein with nine TMDs. TMDs 6 and 7 carry the active site aspartate residues 33,34,35. Upon assembly and maturation of the complex 36, presenilin is cleaved within the large cytoplasmic loop into two fragments, the N-terminal fragment (NTF) comprising TMDs 1-6 and the CTF comprising TMDs 7-9 37. Presenilin endoproteolysis is heterogeneous and occurs in a stepwise manner 38,39 by autoproteolysis 27,33,34,40. The complex can be composed either of presenilin-1 (PS1) or its homolog presenilin-2 (PS2) 41. Likewise, the subunit APH-1 (anterior pharynx-defective 1) 42,43,44, a polytopic membrane protein with seven TMDs, which serves a stabilizing, structural role in the complex, exists as APH-1a or APH-1b variant that are part of separate complexes 45,46. Finally, there is the smallest subunit of the complex, PEN-2 (presenilin enhancer 2) 42,47. Of its two hydrophobic domains, one forms a straight TMD, while the other one adopts a hairpin-like structure embedded in the membrane as two half-helices 48,49. Low-resolution electron microscopy (EM)-structures indicate that the enzyme has substantial conformational flexibility and is compacted upon inhibitor binding 50,51. The identification of three different apo-states in the atomic cryo-EM structure has shown that in particular presenilin together with PEN-2 shows considerable flexibility in the complex 52. The different complexes display differential subcellular locations 53,54. Careful cell biological experiments show that PS1-containing γ-secretase complexes are more broadly distributed within the secretory pathway, whereas PS2-containing γ-secretase complexes localize preferentially to the late endosome/lysosome. These different locations have an impact on the generation of longer Aβ species, as the lysosomal PS2 γ-secretase complexes are responsible for the generation of the bulk of an intracellular Aβ42 pool 54. 

Research over nearly two decades has shown that γ-secretase cleaves numerous other proteins besides APP, and the list of its substrates has grown to more than hundred since the last overview in 2011 55. A general feature that has emerged is that γ-secretase substrates are principally in type I membrane protein orientation, i.e. with an extracellular N-terminus and an intracellular C-terminus, and as an additional key feature have short ectodomains. These are typically generated by ectodomain shedding by proteases such as BACE or in probably most of the cases by metalloproteases of the ADAM (a disintegrin and metalloprotease) family, such as the α-secretases ADAM10 and 17 56. In case of APP, besides C99, the shorter CTFs C89 and C83 are generated by the cleavage of these sheddases 57. However, naturally short substrates, which do not undergo ectodomain shedding such as BCMA (B-cell maturation antigen), have also recently been identified 58. Apart from APP, at least one other major and critical substrate, which stands out is the cell surface receptor Notch1 59,60. Cleavage of Notch1 by γ-secretase is an essential function of the enzyme required for cell-fate decisions during embryonic development and remains also critical in adulthood 61. As demonstrated first for this receptor, the intracellular domain (ICD) generated by γ-secretase cleavage translocates to the nucleus to activate transcription of target genes 62. Similar signaling activities have been reported also for a number of other subsequently identified γ-secretase substrates and have also been suggested for APP 63, although this has remained controversial for this substrate 64. While γ-secretase cleavage can lead to signaling pathway activation, examples for the opposite process have also been identified, where substrate cleavage abolishes signaling events 65,66. The regulation of signaling pathways by the release of biologically active ICDs is thus clearly a vital function of γ-secretase events 67. However, a more general and simple function of γ-secretase could be to clear the membrane from substrate CTFs that are left behind after ectodomain shedding 68. Since an accumulation of these fragments could be toxic as inferable from observations with APP CTFs 69,70,71,72,73, this "membrane proteasome" function could serve to control toxic membrane protein abundance. 

With the discovery of so many γ-secretase substrates it has become clear that the enzyme has multiple biological functions ranging from developmental processes of various organs to functions in the nervous system of the adult brain 55,67. Besides AD, altered γ-secretase function can also be associated with other diseases including most prominently cancer, which has excellently been covered elsewhere 67. It should also be noted here that γ-secretase-independent functions of presenilin have been identified which have recently been reviewed elsewhere 74. 

A precise understanding of how the enzyme recognizes, selects and finally cleaves its substrates is crucial for the development of APP-selective γ-secretase-targeting drugs that should ideally not touch the cleavage of Notch1 and other critical substrates. Understanding this process is an area of active research where important developments have recently been achieved.

## SUBSTRATE RECOGNITION BY γ-SECRETASE

At the very basic level of recognition, substrates of γ-secretase have to locate in the same subcellular compartment as the protease in order to be cleavable. The more restricted localization of PS2-containing γ-secretase complexes to late endosomes/lysosomes thus provides some selectivity in substrate recognition and cleavage as substrates that are targeted to these compartments will preferentially be cleaved by PS2 rather than PS1 54. Once substrate and enzyme encounter each other in the correct compartments, a crucial requirement for the selection of substrates by γ-secretase is that these have short ectodomains typically between 15-30 amino acids in length. The shorter these are the better substrates are cleavable 75,76. Early studies showed that substrates become increasingly less well cleavable when their ectodomain lengths are increased above 50 amino acids 77. This has subsequently been expanded by showing that NCT acts as a gatekeeper controlling whether type I membrane proteins getting in contact with the protease complex have appropriate ectodomain lengths that fit in size underneath the large globular extracellular domain of NCT 76 (**Fig. 2**). In agreement with such a substrate size control function of NCT are observations that antibodies against the NCT ectodomain can block substrate binding and catalysis 78,79. Although experimentally not yet shown, it is likely that bound antibodies sterically hinder substrate access to the enzyme. This could occur either directly or by inducing a conformational change of the NCT ectodomain towards the membrane-embedded part of the complex thereby interfering with substrate recruitment. Flexibility of NCT has been inferred by lower resolution EM studies 50,51 and is supported by molecular dynamics computer simulations 80,81. 

**Figure 3:**
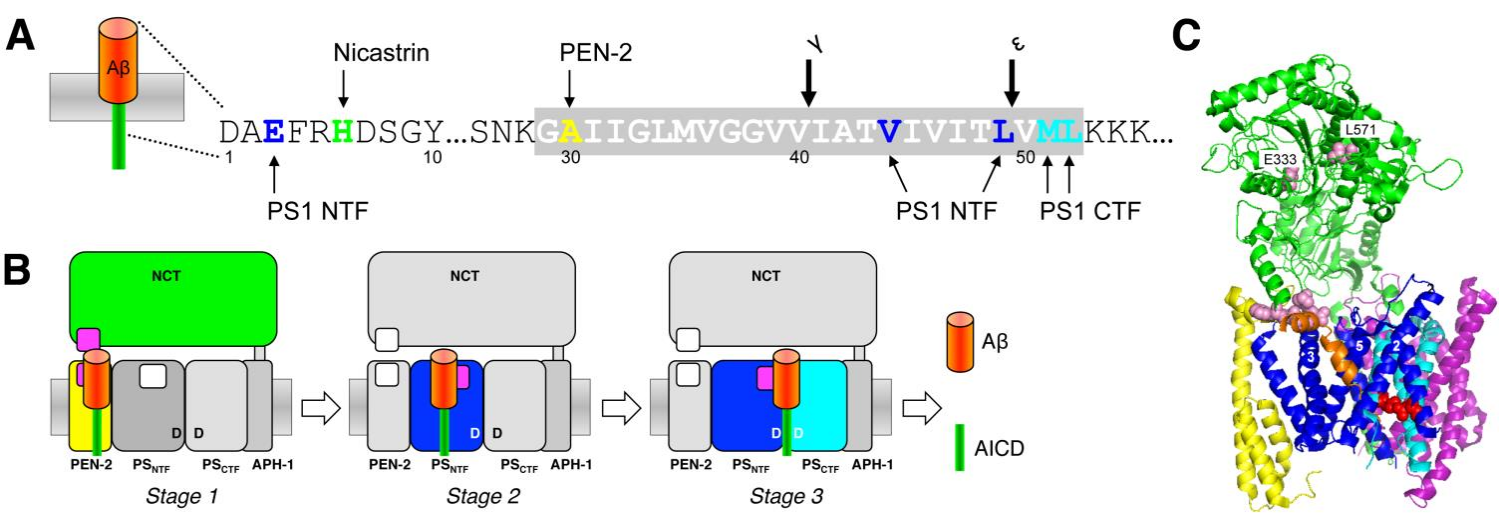
FIGURE 3: Substrate recruitment of C99 by γ-secretase. **(A)** Schematic representation of the most prominent C99 residues interacting with γ-secretase subunits as determined by site-directed photocrosslinking using the unnatural amino acid *p*-benzoyl-phenylalanine. **(B)** Model depicting the stepwise translocation of C99 from exosites (purple) in NCT and PEN-2 (stage 1) and the PS1 NTF (stage 2) to the active site (stage 3). **(C)** Structure of γ-secretase (5FN3) with a co-isolated α-helix (orange), which might represent a substrate-mimic. Red spheres depict the active site aspartate residues. Light pink spheres represent candidate sites for substrate interactions with NCT. Although not visible in this view, L571 is less buried than E333. Numbers indicate TMDs of the PS1 NTF that surround the substrate-mimicking α-helix.

Following this initial recognition step, in which type I membrane proteins with too large ectodomains are prevented from recruitment to the protease by steric hinderance 76, substrates gain access to the catalytic site in a stepwise-manner by sequential interactions with exosites, substrate binding sites outside the active site 82. Photoaffinity-labeling studies with C99 using site-specifically introduced *p*-benzoyl-phenylalanine as crosslinkable amino acid identified the PS1 NTF as major substrate-binding site. Prominent contact points of C99 occurred in the cleavage site region and included among other residues V44 between the γ- and ζ-sites and around the ε-cleavage sites L49 that contact the PS1 NTF as well as residues M51 and L52 that predominantly contact the PS1 CTF 82 (**Fig. 3A**). In addition, NCT, PEN-2 and the PS1 NTF were identified as exosite-bearing γ-secretase subunits. Here, the most prominent contact points of C99 were H6 with NCT, A30 with PEN-2 and E3 with the PS1 NTF (**Fig. 3A**). Furthermore, as demonstrated for C99 by the photocrosslinking experiments, exosites in NCT and PEN-2 mediate the first interactions with γ-secretase (stage 1). Upon release from these sites, C99 binds to exosites in the PS1 NTF (stage 2). Following this stage, C99 can engage the catalytic site for cleavage (stage 3) (**Fig. 3B**). Binding of C99 to NCT at stage 1 is consistent with earlier findings that suggested an active role of this subunit in substrate recognition as a substrate receptor 83. Although the contact points of C99 in the enzyme are currently not known, the initially proposed model that NCT interacts with the free N-terminus of substrates via an electrostatic interaction with E333 in its DAP domain (DYIGS and peptidase; residues 261-502) 83 has been controversial 76,84,85 and ultimately seems unlikely (**Fig. 3C**). As seen in the structure, E333 located in the large lobe cannot directly be accessed by a substrate because it is covered by a "lid" region in the small lobe, which would need to be displaced by a conformational change involving rotation around a pivot 30,86. Since mutations of lid and pivot residues did not interfere with substrate cleavage 87, the proposed mechanism cannot apply and suggests that the substrate may contact NCT at another region. Interestingly, L571, another residue of the large lobe in α-helix 16 is less buried than E333 and was found to be critically required for substrate binding and catalysis 79, indicating that the substrate´s N-terminal extracellular domain may contact the protease in this region (**Fig. 3C**). Another candidate interaction site could be the loop of residues 240-244 of NCT, which is in close proximity to a co-purified potential substrate-mimic that adopts a kinked transmembrane α-helix in the class 1 apo state of the enzyme 52 (**Fig. 3C**). For PEN-2, the candidate-binding region of C99, which interacts with the extracellular juxta-membrane region of C99 including the N-terminal TMD end, is likely its corresponding extracellular juxtamembrane region starting from the C-terminal end of TMD 2. Whether C99 interacts with NCT or PEN-2 at stage 1 sequentially or simultaneously is not yet clear. 

It is highly likely that the substrate movements of C99 from the exosites encountered in stages 1 and 2 to the active site are associated with substantial conformational changes of the PEN-2 and presenilin TMD core of the complex. Lateral movement of PEN-2 towards the active site, potentially together with NCT and rearrangements of PS1 NTF TMDs 2-6 could translocate exosite-bound substrates to the active site. Based on the cryo-EM class 1 structure with the kinked substrate mimic, substrate entry to the active site may occur through the cavity formed by TMD2, TMD3 and TMD5 52 (**Fig. 3C**). The PS1 hydrophilic loop 1 may play an important role in this process by allowing movements of TMD2, which like TMD6 is highly dynamic 30,48,52. This would be consistent with mutational analysis that implicated this region in substrate binding 88 and supported by the clustering of many FAD mutations in this loop. Ultimately, as suggested by mutational analysis, a final substrate selection step likely takes place close to the catalytic aspartates and involves the GxGD-motif in TMD7. The conserved glycines are highly critical for γ-secretase activity 89,90 and L383 of PS1 at position x of the motif in PS1 provides substrate selectivity of APP over other substrates, such as Notch1 or CD44, that are not cleavable with certain substitutions of this residue 91,92. This suggests that the GxGD motif is a critical region for the proper exposure of the substrate scissile bonds to the catalytic residues. Substrates not meeting the steric requirements may fall off from the enzyme at this stage. Similar observations have been made by mutational analysis of the conserved PAL motif, which is also located in close vicinity to the active site 93,94.

## SEQUENTIAL CLEAVAGE OF THE APP TMD BY γ-SECRETASE

**Figure 4:**
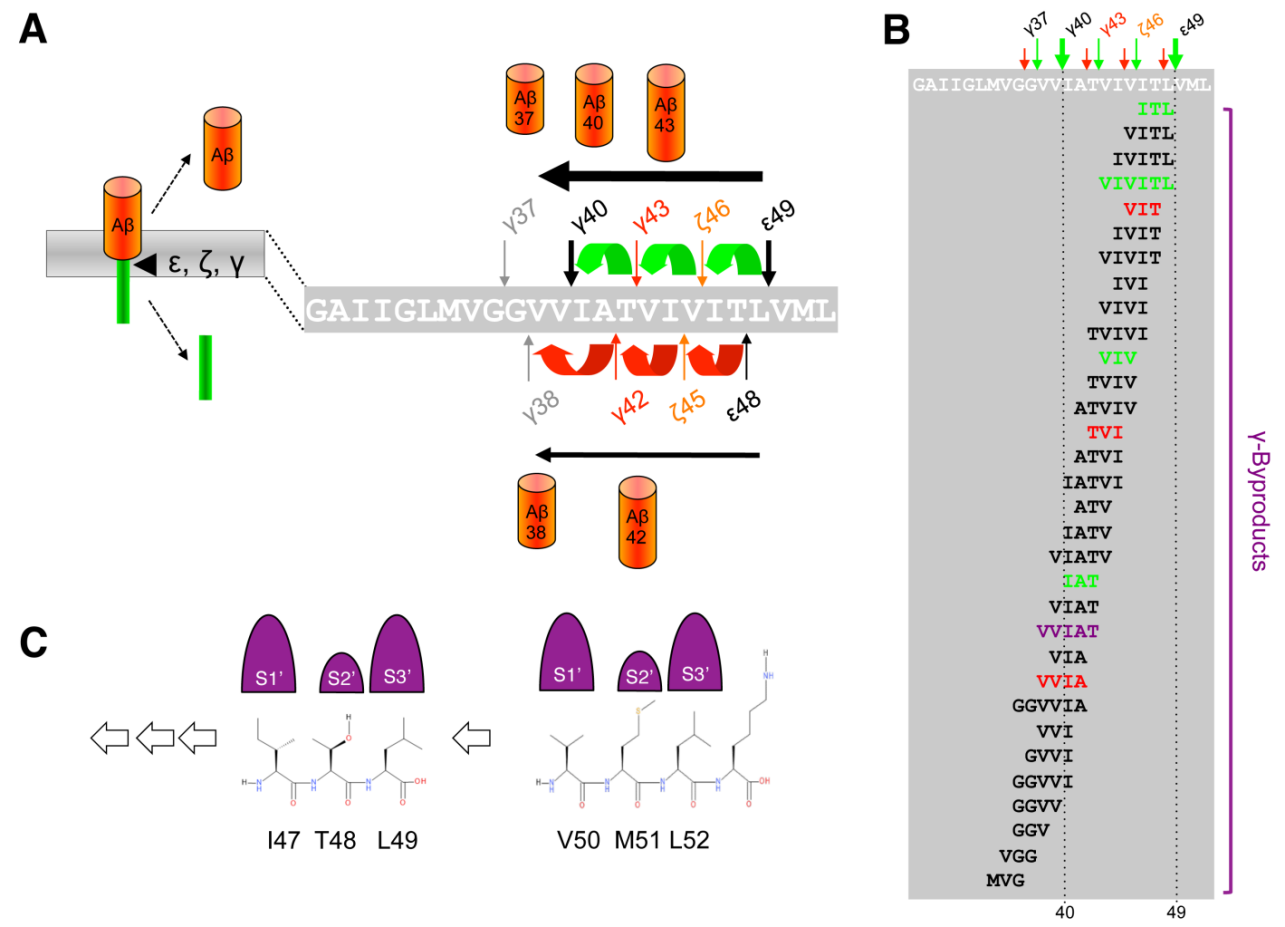
FIGURE 4: Stepwise cleavage model of APP. **(A)** The two product lines leading to the generation of Aβ40 and Aβ42 are depicted. In the major product line Aβ40 is generated by consecutive tripeptide-releasing cleavages (green) at the ε-49, ζ-46 and γ-43 sites. In a minor product line, Aβ42 is generated in a similar manner by consecutive cleavages (red) at the ε-48 and ζ-45 sites. **(B)** Besides γ-byproducts from the two main product lines (green and red) including a hexapeptide resulting from direct cleavage of Aβ49 to Aβ43, multiple additional minor peptides have been identified suggesting multiple product line crossings. The pentapeptide resulting from cleavage of Aβ43 to Aβ38 is indicated in purple. **(C)** Sequential cleavage continuously requires sterically compatible interactions of P2´ residues with the S2´ enzyme subsite of γ-secretase; major product line shown. The adjacent P4´ residue K53 is additionally presented for the comparison of side chain proportions.

When C99 has reached the active site region, γ-secretase generates Aβ by consecutive intramembrane cleavages at the ε-, ζ-, and γ-cleavage sites (**Fig. 4A**). Following an initial endoproteolytic cleavage at the ε-49 or ε-48 cleavage sites 95,96,97,98, γ-secretase cleaves the remaining membrane-bound long Aβ counterparts by stepwise carboxyterminal trimming 99,100,101. In the major pathway, Aβ40 is produced in a product line starting from Aβ49 via the intermediates Aβ46 and Aβ43. Aβ42 is generated likewise in a minor product line starting from Aβ48 via the intermediate Aβ45. Cleavages in the two lines can also continue further to the shorter species Aβ37 or Aβ38, respectively. 

Since the γ-cleavages occur in the middle of the TMD, while the ε-cleavages close to the cytosol border of the TMD, the C-termini of Aβ40/42 generated by γ-cleavage and the N-termini of AICD by ε-cleavage are distinct. It had thus initially been unclear whether ε-cleavage would precede γ-cleavage or the other way round or whether both cleavages may occur independently. A landmark discovery that provided a first evidence for a stepwise cleavage was the detection of C-terminally elongated Aβ species such as Aβ43, Aβ45/46, and Aβ48/49 in lysates of cultured cells 99,101,102. Since corresponding N-terminally elongated AICD species (AICD-γ43 or AICD-ζ45/46) were also not found for the longer Aβ43 and Aβ45/46 species, it was hypothesized that ε-cleavage at the ε48/49 sites is followed by sequential cleavages proceeding to the N-terminus at every third or fourth residue, thereby resulting in generation of Aβ40 and Aβ42 99. Thus, assuming an α-helical conformation of the C99 TMD, small three to four amino acid peptides are produced every time the cleavage proceeds along the helix 99 (**Fig. 4A**). Accordingly, following the release of AICD-ε49 and AICD-ε48, the two Aβ production pathways outlined above in which Aβ40 is produced from Aβ49 and Aβ42 from Aβ48 were proposed 99. 

Using a cell-free γ-secretase cleavage assay, in which cleavage of a recombinant C99 substrate by detergent-solubilized γ-secretase is directly analyzed, the putative tri- or tetrapeptides between the ε-, ζ- and γ-cleavage sites (G_37_GVVIATVIVITL_49_) were identified by liquid chromatography tandem-mass spectrometry 100. Thus, ITL, VIV, and IAT peptides, of the Aβ40 product line, as well as VIT, TVI, and VVIA peptides of the Aβ42 product line were detected as “γ-byproducts” 103 of the cleavage reaction (**Fig. 4B**). In agreement with the sequential γ-cleavage model, the peptides were generated in a time-dependent manner 100. Further strong evidence for this model was obtained by successfully measuring these small peptides also inside cultured cells 104 and *in vivo* in the brain of PS1 I213T knock-in mice overexpressing Swedish APP 103. Interestingly, also a VVIAT pentapeptide resulting from a cleavage of Aβ43 to Aβ38 was detected both in the cell-free γ-secretase cleavage assay and in cultured cells 104 (**Fig. 4B**). Notably, ∼40% of Aβ38 was derived from Aβ43 in cells showing that Aβ38 can not only have Aβ42 as a precursor and that the Aβ40/42 product lines can overlap. In line with these findings, it could further be shown that both Aβ42 and Aβ43 itself can serve as γ-secretase substrates and be cleaved to Aβ38 104. Alternative minor production pathways with additional product line crossings were identified in other studies 105,106. Altogether, the detailed analyses of the sequential cleavage mechanism of APP showed that it is much more complicated than it was initially considered (**Fig. 4B**). A critical implication of the sequential cleavage model for AD pathogenesis is that a processivity impairment in the Aβ product lines will lead to increases in the pathogenic Aβ42/43 species and an intracellular accumulation of longer Aβ species in membranes, which may also be pathologically relevant in AD for the origin of neuritic plaques as a consequence of neuronal loss 107. 

The mechanism by which γ-secretase releases tripeptides in the sequential cleavage of C99 to Aβ was worked out by a recent study showing that γ-secretase has three distinct amino-acid-binding pockets in the active site region corresponding to the S1´, S2´ and S3´ subsites through which the enzyme forms a stable enzyme-substrate scission complex 108 (**Fig. 4C**). Fitting of the P1´- P3´ residues into these pockets brings the substrates into position for each catalytic cycle of the sequential cleavage. The S2´ pocket is smaller than the S1´ and S3´ pockets, which imposes steric requirements on the P2´ site of C99 and the ensuing Aβ substrates (**Fig. 4C**). It is currently unknown whether other substrates of γ-secretase follow the sequential cleavage model. However, this is not unlikely, since longer Aβ-like peptides such as APL1β28 produced from APLP1 or Nβ25 produced from Notch1, can serve as γ-secretase substrates and be cleaved *in vitro* into APL1β25 and Nβ21, respectively, indicating sequential cleavage mechanisms as well 104. In addition, multiple cleavage sites have been identified in a number of substrates now that may possibly relate to consecutive cleavages along one or more product lines as well 109. 

The molecular properties of substrates, which are recognized by γ-secretase differentiating them from nonsubstrates, are largely unknown. Since cleavages of C99 and Notch1 are kinetically extremely slow with very low turnover numbers *k*_cat_ 76,110, it is likely that conformational flexibility of the substrate, in particular TMD helix dynamics, plays an important role to find the conformations that allow productive accommodation into the enzyme at the exosites and/or the active site 111,112. Indeed, insertion of helix stabilizing and destabilizing residues in the cleavage domain has an inhibiting or promoting impact, respectively, on the cleavability of C99 113,114.

**Figure 5:**
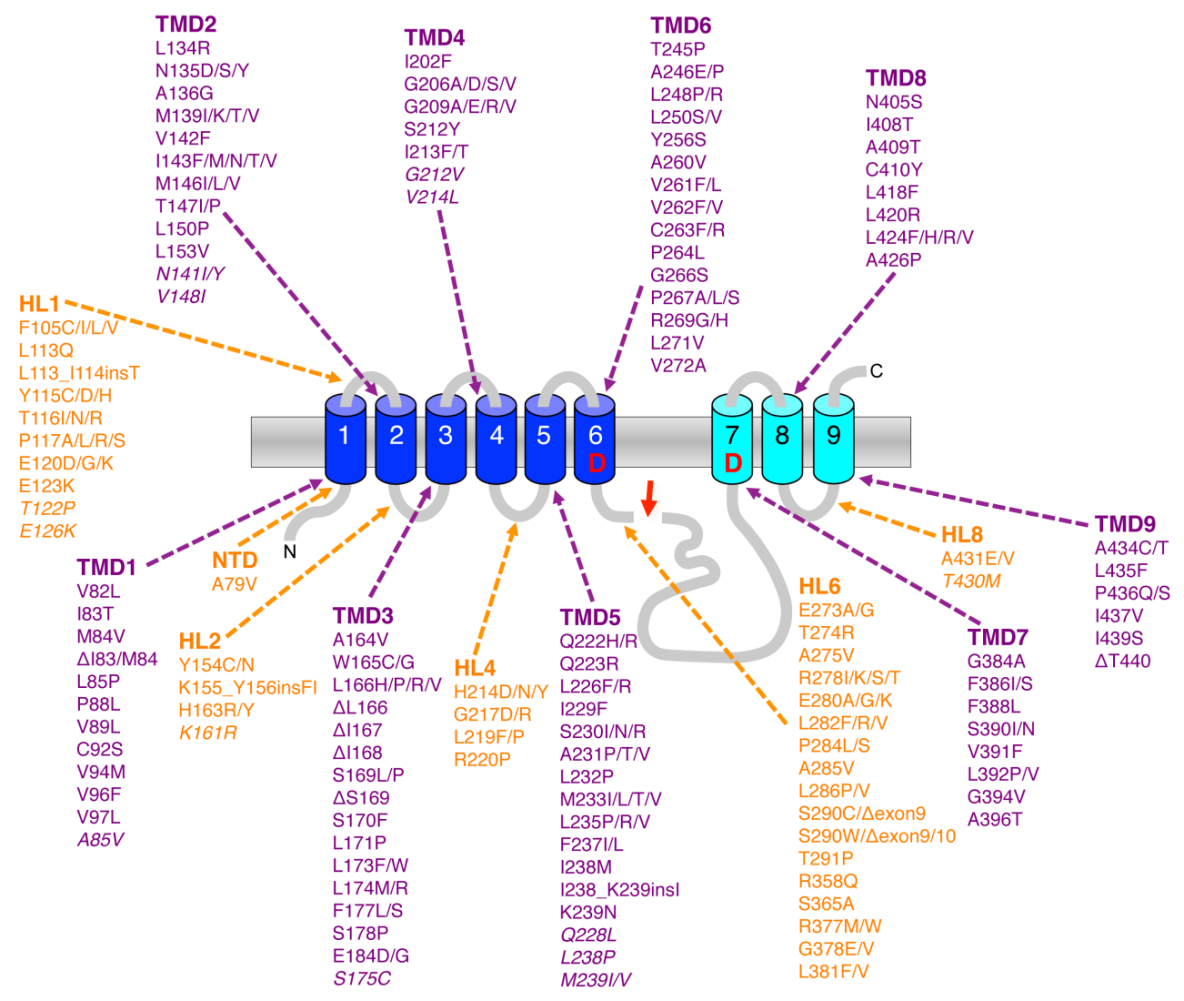
FIGURE 5: FAD mutations in presenilin. Schematic representation of the nine TMD structure of presenilin in its cleaved form with the NTF (blue) and CTF (cyan). Pathogenic presenilin mutations (http://alzforum.org/mutations/) are found in all TMDs and in some of the HLs. The compared to PS1 less frequent PS2 FAD mutations are represented in italics. The red arrow indicates the site of endoproteolysis.

## MECHANISMS OF PRESENILIN AND APP FAD MUTATIONS UNDERLYING THE GENERATION OF PATHOGENIC Aβ SPECIES

**Figure 6:**
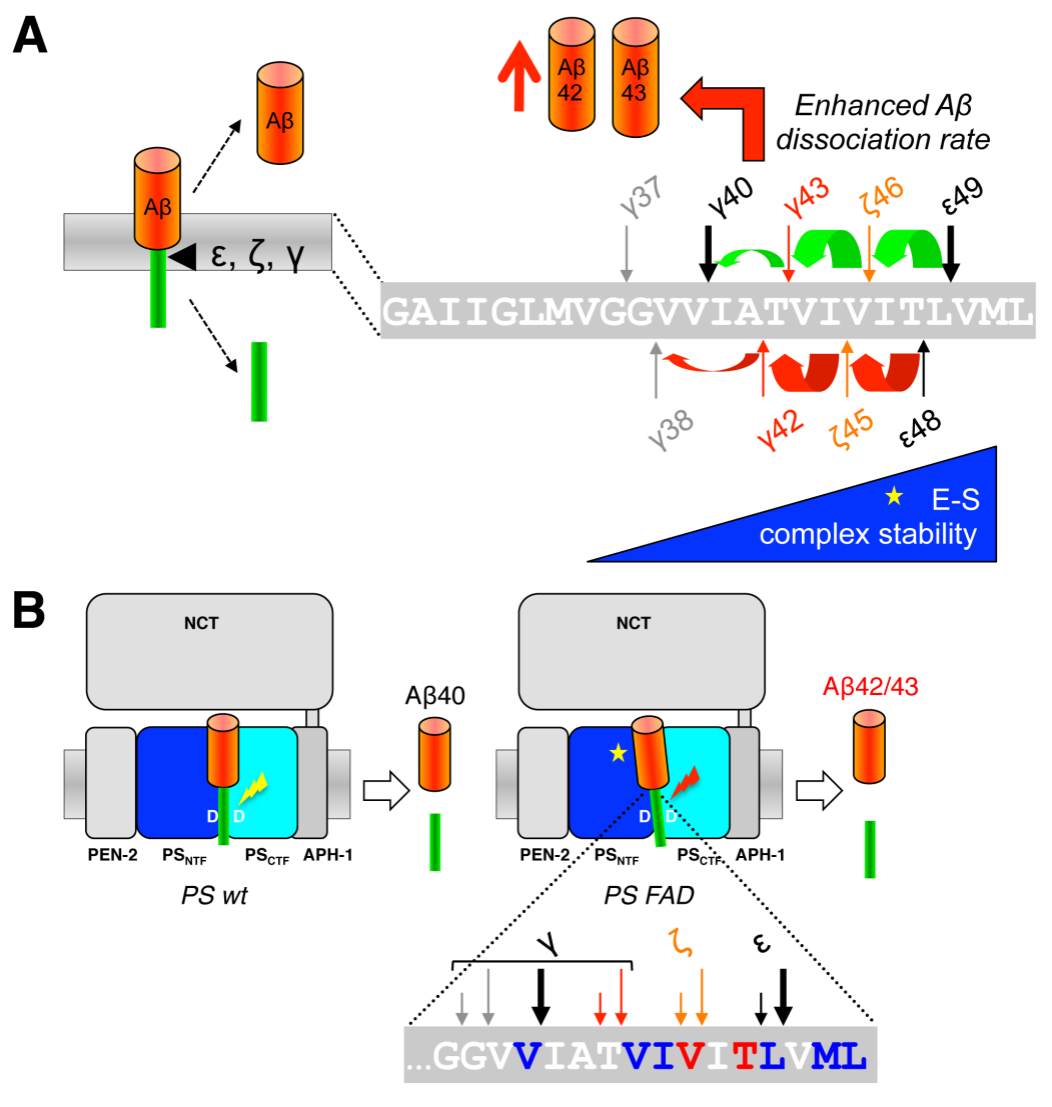
FIGURE 6: Mechanism of presenilin FAD mutations. **(A)** Model depicting impaired processivity in the Aβ40 and Aβ42 product lines causing relative increases in the generation of the longer Aβ species Aβ42 and Aβ43. Structural instability of FAD mutant presenilin leads to impaired processivity manifested by faster dissociation rates and premature release of long Aβ such as Aβ42 from the enzyme. **(B) **Presenilin FAD mutations cause a mispositioning of C99 as shown by altered interactions of the substrate cleavage domain with the enzyme. Residues that were identified to show increased or decreased interactions with two different PS1 FAD mutants are shown in red and blue, respectively. Yellow asterisk indicates a FAD mutation.

By far the most mutations associated with FAD are found in the PS1 gene. To date over 210 pathogenic mutations have been identified covering ~25% of the residues (**Fig. 5**). Almost all of them are missense mutations that localize all over the protein clustering in the TMDs, in hydrophilic loop (HL) 1 and HL6 N-terminal to the endoproteolytic cleavage site within exon9. Mutations in PS2 show a similar broad distribution over the molecule but are much less frequent and have a later disease onset that is likely due to the lower expression of PS2. Although it had already been shown shortly after the discovery of presenilins that the mutations change the Aβ42/40 ratio 15, the mechanism(s) behind this phenomenon has remained obscure for a long time and only recently become more clear. Following the endoproteolytic cleavage at the ε-site, the carboxy-terminal trimming of the 42-product line becomes impaired such that the enzyme cannot efficiently convert Aβ42 to Aβ38 115,116,117 (**Fig. 6A**). As a consequence, the generation of Aβ38 decreases causing an increase in the Aβ42/38 ratio. In addition, this leads to a relative increase of the generation of Aβ42 over that of Aβ40 and thus to an increased Aβ42/Aβ40 ratio. Likewise, trimming can also become impaired in the 40-product line manifesting in increased Aβ43/40 ratios. Depending on the mutation, these principal effects can also occur in combination, leading to an increased ratio of Aβ42/43 to Aβ40. Why these processivity changes occur for presenilin FAD mutations has been enigmatic and only recently convincing explanations could be offered. Thus, it could be shown that PS1 FAD mutations display an increase in the dissociation rate of Aβ42 from the enzyme 104. In line with these findings, it was next found that FAD mutations destabilize the enzyme and as a consequence also the enzyme-substrate interactions 118. Enzyme destabilization affected the initial enzyme-C99 interaction leading to impaired endopeptidase cleavage at the ε-site and continued to also weaken the interactions with the enzyme of the subsequently generated Aβ substrates. In addition to these findings, it was found that interactions of C99 with γ-secretase are altered by FAD mutants in the substrate cleavage domain, thereby changing the positioning of the substrate in the active site region 82 (**Fig. 6B**). Consistent with these recent results, earlier studies had suggested that FAD mutations affect the topography of the active site as shown by reduced inhibitor potencies 119,120,121 and altered interactions with active-site targeted inhibitors 122. The model emerging from these studies that puts all observations together is that FAD mutations in presenilin cause structural alterations, which can, depending on the particular mutation, destabilize the enzyme to various extents. This leads to altered C99 binding and labile interactions of the subsequently generated longer Aβ species which dissociate faster from the enzyme than in the wild-type (wt) situation. 

**Figure 7:**
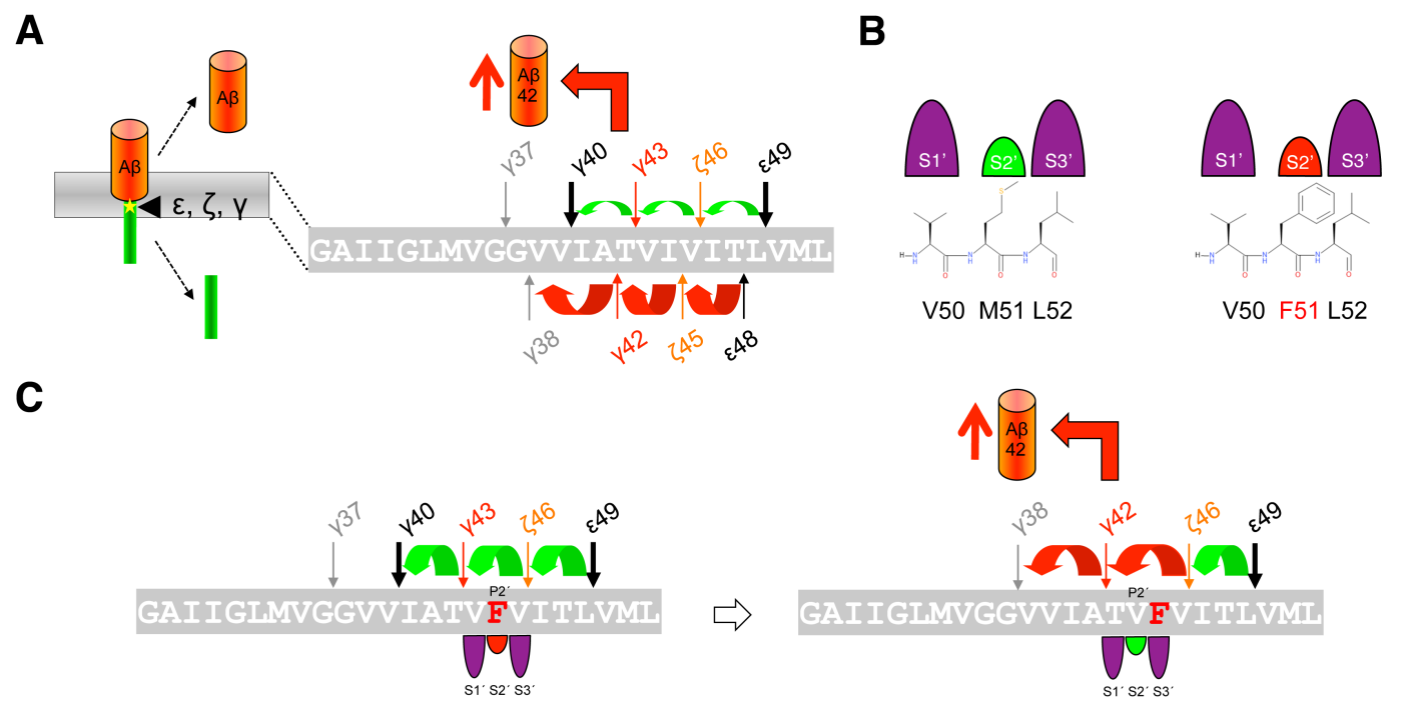
FIGURE 7: Mechanism of APP FAD mutations.(A) Model depicting the shift in the Aβ40 and Aβ42 product lines by APP FAD mutations thereby causing relative increases in the generation of the longer Aβ42 species. Yellow asterisk indicates a FAD mutation. **(B) **Product line usage is governed by interactions with the S2´ enzyme subsite of γ-secretase. Sterically demanding aromatic amino acids at the P2´ position of C99 such as in the synthetic M51F mutant clash with the S2´ subsite of the Aβ40-line. **(C)** Pathway block by a clash of the aromatic P2´ with the S2´ subsite of the Aβ40-line in the FAD-associated I45F mutant causes a shift to the Aβ42-product line. Since this mutant does not alter the initial ε-site cleavage, Aβ42 is most likely generated from Aβ46.

The mechanism of APP FAD mutations is less clear than that of the FAD mutations in presenilin described above. It had been reported that FAD mutations located in the γ-secretase cleavage region behave differently from those in presenilin that typically affect the efficiency of the conversions of Aβ42 to Aβ38 and of Aβ43 to Aβ40, and unlike these, cause a shift to the Aβ42 product line 115 (**Fig. 7A**). When this product line is entered, not only increased amounts of Aβ42 are generated. Remarkably, also increased Aβ38 levels, often comparable to those of Aβ42, have been observed 115, although not all studies showed consistent results 99,106,115,118,123,124,125. Considering that the affinities of the sequentially generated Aβ species for the enzyme are decreasing from longer to shorter peptides 118, the accumulation of Aβ38 to similar levels as Aβ42 is surprising. Although Aβ38 can also be generated from Aβ43 104, this should occur only to a very minor extent since the Aβ40 product line is not the preferred product line of APP FAD mutations. How APP FAD mutations cause an increase in the Aβ42/40 ratio can therefore not yet fully sufficiently be explained by these observations and additional mechanisms besides ε-site shifts have to be at play. Indeed, it has been demonstrated that the nature and the location of the FAD mutation will dictate which product line is selected 108. It could be shown that the three residues locating C-terminal to the cleavage sites, i.e. residues P1´ - P3´, need to fit to the apparently large S1´, the small S2´, and the large S3´ subsite pockets of the en-zyme. Thus, the presence of aromatic amino acids at the P2´ position will clash with the small S2´ pocket, thereby blocking the respective Aβ40 or Aβ42 product line (**Fig. 7B**). For example, a phenylalanine occurring at the P2´ site of the γ-43 cleavage site as in the I45F FAD mutant, will cause a shift from the Aβ40 product line to the Aβ42 product line because the bulky phenylalanine will better accommodate to the large S3´ pocket of the γ-42 cleavage site (**Fig. 7C**). Interestingly, this happens without the typical ε-site shift 108. However, the other so far known FAD mutant with a phenylalanine mutation, the V46F mutant, is not causing a product line block as the phenylalanine substitution does not clash with the small S2´ pocket in either of the two pathways. In this case, the increased Aβ42/40 ratio is caused by the product line shift at the ε-site. 

Taken together, while presenilin FAD mutations display reduced carboxy-terminal trimming within the two product lines and/or product line shifts, the APP FAD mutations seem to combine product line shifts with substrate side chain compatible occupancy of the S1´- S3´ pockets. A problem that is not yet solved with the currently available models is the generation of Aβ38. This species is generated for a number of PS1 FAD mutants at levels comparable to the wt enzyme and in increased amounts for at least some APP FAD mutants, although it should not be generated when Aβ42 and Aβ43 dissociate faster from the enzyme due to destabilized enzyme-substrate interactions in the mutant case 118. Although exciting research of the past few years has thus provided increasing mechanistic insights, the models derived from these studies are still too simple to explain all experimental observations at the mechanistic level. 

## THE PRESENILIN LOSS OF FUNCTION HYPOTHESIS OF AD

Although the genetics strongly suggests that Aβ is central to AD pathogenesis, it has been challenging that neurodegeneration is not observed in transgenic mouse models that produce and extracellularly deposit aberrant amounts of pathogenic Aβ species by the expression of FAD mutant APP and/or presenilin variants (as an example see 126). On the other hand, neurodegeneration has been observed in conditional PS1 knockout in mice in the absence of pathogenic Aβ production 127. This has led to the hypothesis that a loss of presenilin function in cleaving crucial physiological substrates such as Notch1 is responsible for (F)AD rather than an absolute increase of total Aβ or a relative increase of Aβ42 128. An initially perplexing finding which seemed to support the "presenilin hypothesis" has been the biology of the PS1 L435F mutant, which causes early onset FAD although the mutant is virtually inactive and does not support the generation of Aβ40 and Aβ42 129. To explain the phenotype of this mutant, it was suggested that wt and FAD mutant presenilin form a dimeric complex in which the mutant could exert a dominant negative effect on the wt protein forcing it into a pathogenic Aβ42-generating conformation 129. However, it has now been found that the PS1 L435F mutant generates and deposits the pathogenic Aβ43 species 130, which had been omitted from the Aβ analysis in the previous studies 131,132. Importantly, this and other similar FAD mutants were shown to retain the capability to generate the pathogenic Aβ43 species in the absence of endogenous presenilins 131,132. Potential trans-dominant effects of mutant presenilins on the wt protein that have been reported in the literature 133,134 can therefore not play a decisive role in the generation of pathogenic longer Aβ species and AD pathogenesis. 

While presenilin FAD mutants can impair the cleavage of many substrates and thus potentially block or alter signaling pathways mediated by their ICDs, this seems unlikely to play a major role *in vivo*. The analysis of γ-secretase activity in human brain samples of PS1 FAD cases has shown that AICD formation is not affected whereas the carboxy-terminal processivity defects leading to increase Aβ42/40 ratios persist 117. These data strongly suggest that potential effects on the "signaling cleavage" are compensated by the remaining wt PS alleles. A recent study investigating a total of 138 PS1 FAD mutations could not find a correlation with increased Aβ42 ratios and the age of onset while the vast majority of the mutants showed strongly reduced total γ-secretase activity 135. This has been taken as another argument in favor of the presenilin hypothesis. However, the Aβ40 and Aβ42 levels and corresponding Aβ42/40 ratios of many PS1 FAD mutants that are well-characterized in cell-based assays were not recapitulated well in these assays and the pathogenic Aβ43 species had not been measured in the study. Moreover, predicting the age of onset of mutations from γ-secretase activities measured in cell-free *in vitro* assays using purified γ-secretase preparations in which loss of function effects are known to be more strongly pronounced 136 is problematic as this system differs dramatically from the heterozygous situation in FAD patient brain. Importantly, if loss of γ-secretase activity by presenilin mutations should be causative for AD, then haploinsufficiency of other γ-secretase subunits should also cause the disease. However, nonsense mutations that have been identified in PS1, NCT and PEN-2 are implicated in the cause of the rare skin disease acne inversa and not AD 137. Finally, it is obvious that all types of APP FAD mutations as well as the protective Icelandic mutation or the presence of AD in Down syndrome patients with an APP gene triplication are not explainable by the presenilin hypothesis. Taken together, balancing the available evidence, the support of the amyloid cascade hypothesis remains overwhelming, whereas the problems of the presenilin hypothesis persist.

## FAILURE OF γ-SECRETASE INHIBITION IN CLINICAL TRIALS

Since the amyloid cascade hypothesis predicts that lowering Aβ should be beneficial for AD, γ-secretase has been an obvious and major drug target. Thus, to inhibit its enzymatic activity and blocking Aβ generation, γ-secretase inhibitors (GSIs) were developed 138,139. They fall into two principal classes, transition-state analogue (TSA) inhibitors, such as the prototypic L-685,458 140 and related compounds as well as non-TSA compounds. The former compounds, which target the catalytic site of γ-secretase were the first highly potent inhibitors identified. Non-TSA inhibitors such as DAPT 141 or LY450139 (semagacestat) 142 are considered to bind nearby the active site. Unfortunately, since such pan-GSIs inhibit both Aβ and Notch ICD (NICD) production, severe side effects were observed when administered* in vivo*, mainly due to disturbance of Notch signaling 143,144,145,146. While this is unwanted for AD therapy, treatment of certain cancers may benefit from Notch pathway inhibition by GSIs 147. Several non-TSA GSIs were reported to have less inhibitory effects on NICD generation at a concentration range at which they sufficiently reduce Aβ secretion. Such Notch-sparing GSIs, as for example the promising candidate BMS-708163 (avagacestat) 148, were thus considered as improved AD therapeutics that should minimize side effects due to inhibition of Notch signaling. Altogether, many clinical studies have been conducted using non-TSA GSIs for the therapy of AD and/or are ongoing for cancers. Disappointingly, all of the AD clinical trials failed including a large phase 3 trial of semagacestat 149. Even worse is that this trial and that of avagacestat 150 not only ended up with severe adverse effects but also with aggravated cognitive decline in patients. As semagacestat is a non-selective GSI and since follow-up studies suggested that the APP over Notch selectivity window of avagacestat was too narrow if present at all 115,151, the observed side effects can likely be attributed to the inhibition of Notch signaling. There has been no definitive answer yet on the cause of the aggravation of cognitive decline.

## PSEUDOINHIBITION OF γ-SECRETASE BY SEMAGACESTAT AND RELATED GSIs

**Figure 8:**
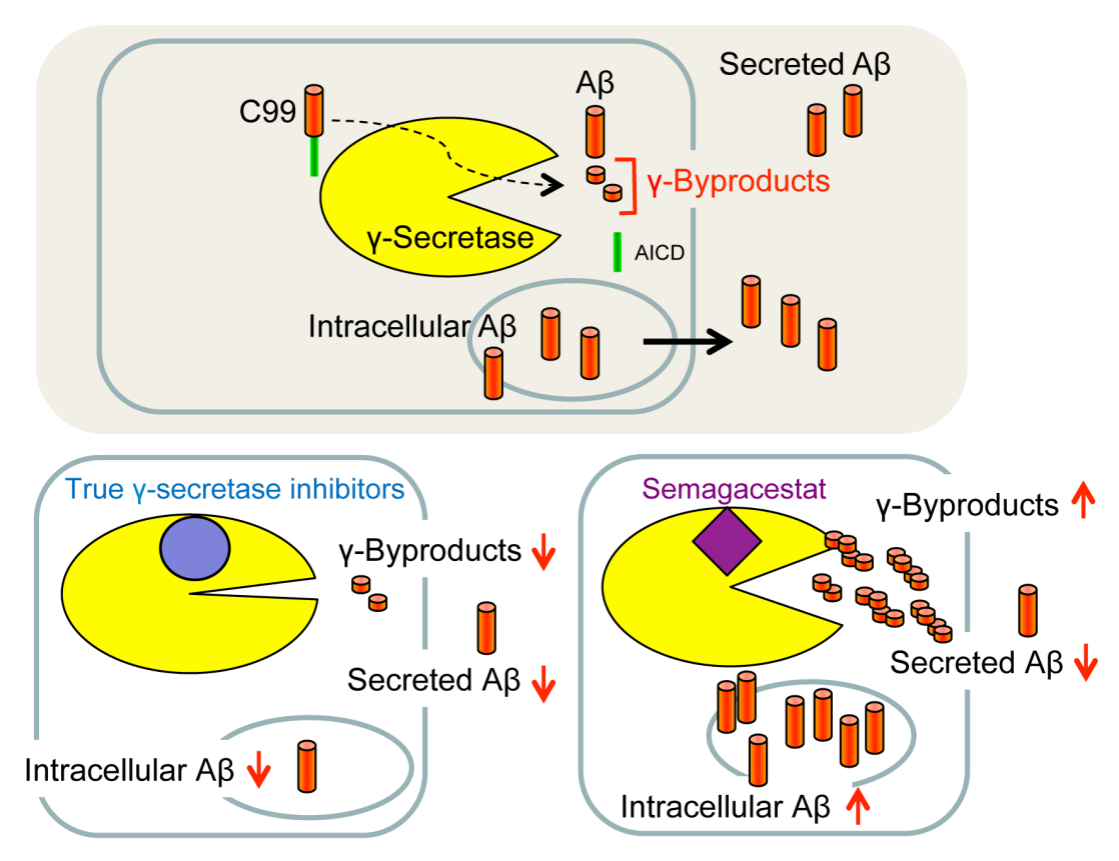
FIGURE 8: Pseudoinhibiton of γ-secretase by semagacestat. Cleavage of C99 by γ-secretase causes the generation of secreted and intracellular Aβ pools as well as γ-byproducts from carboxy-terminal trimming of the C99 TMD. Unlike TSA-GSIs such as L-685,458, semagacestat causes an intracellular accumulation of long Aβ species and byproducts demonstrating a pseudo-inhibition of γ-secretase by this and related compounds.

Using Aβ secretion as readout, GSIs were principally identified by high-throughput screening of small molecule libraries for compounds capable of lowering Aβ in conditioned media of cultured cells. However, since Aβ secretion is the result of several steps including its generation, liberation from membrane, trafficking, intracellular degradation and secretion, the levels of secreted Aβ do not necessarily accurately reflect the activity of γ-secretase. A further complicating and puzzling issue has been the observation that some GSIs, including DAPT, consistently increased the amounts of long intracellular Aβ species such as Aβ46 in APP-overexpressing cultured cells when used at concentrations that inhibit the formation of secreted Aβ 99,101,102. The reasons had been unclear at the time, and clinical trials were performed without addressing this issue, although accumulation of such longer Aβ forms might be toxic to membranes. Considering that the γ-byproducts that are released by the sequential γ-secretase cleavages are not secreted and thus could serve as a more direct indicator of γ-secretase activity than secreted Aβ, the mechanism of action of non-TSA GSIs used in clinical trials was re-investigated by measuring their levels as readout 103. Surprisingly, non-TSA GSIs, including semagacestat and avagacestat, did not decrease but rather increased the levels of γ-byproducts inside neurons derived from human iPS cells 103. Along with the increased γ-byproducts levels, also an accumulation of long Aβ such as Aβ45 and Aβ46 was found inside neurons, although semagacestat and avagacestat decreased secreted Aβ. In contrast, L-685,458 decreased the levels of γ-byproducts and intracellular Aβ acting equivalently to a γ-secretase loss of function. The increased levels of γ-byproducts and the intracellular accumulation of long Aβ forms suggests that non-TSA GSIs, such as semagacestat and others, are in fact pseudoinhibitors of γ-secretase (**Fig. 8**). Apparently, these GSIs allow γ-secretase to initially cleave C99 but then γ-byproducts and/or inefficiently processed long Aβ intermediates may not efficiently be released from the enzyme and block access of further substrate eventually leading to a reduction of secreted Aβ as a secondary event. Interestingly, it was also found in this study that the γ-byproducts accumulate in the membrane 103. This observation indicates that clearing the γ-byproducts from the membrane by facilitating their release into the hydrophilic space may be an additional function of γ-secretase. Semagacestat and other non-TSA GSIs may thus not inhibit the proteolytic activity of the enzyme but rather such a clearing function. One should note, however, that accumulation of γ-byproducts and long Aβ intermediates upon γ-secretase inhibition with DAPT has not been observed in reconstituted γ-secretase assays using detergent-solubilized enzyme 100,152. This may be because such assays have only a simple and limited lipid composition compared to membrane-based cell-free assays with crude membrane fractions containing native γ-secretase 153,154 in which accumulation of γ-byproducts is observed 103, and/or because residual detergent present in these assays keeps the γ-byproducts soluble. Further analysis is necessary to elucidate whether γ-secretase has such a putative γ-byproduct clearance activity, and if so, whether and how a potential malfunction could be involved in AD pathogenesis as intracellular buildup of γ-byproducts or in particular of longer Aβ in neurons may potentially cause membrane toxicity.

## TARGETING γ-SECRETASE SAFELY

Unfortunately, the failure of GSIs in major clinical trials since 2010 caused a discouragement of pharmaceutical companies that lead to a stop in drug development for this AD target. However, GSIs that went into the trials were developed when our understanding of the many functions of γ-secretase and of its cleavage mechanism was very limited. Clearly, as it turned out, pan-inhibition of γ-secretase is highly problematic due to inhibition of critical substrates other than APP C99, potentially toxic C-terminal substrate fragment accumulation and Aβ rebound-effects from accumulated C99 155 as well as cross-reactivities with signal peptide peptidase and related protease family members 139. In addition, non-TSA GSIs that have been in clinical trials may paradoxically increase potentially toxic longer Aβ intermediates and γ-byproducts 103.**

Rather than GSIs, γ-secretase modulators (GSMs) could be beneficial for AD. As shown first for a subset of NSAIDs (non-steroidal anti-inflammatory drugs), these compounds selectively lower the generation of Aβ42 while concomitantly increasing that of Aβ38, without affecting ε-site cleavages of γ-secretase substrates including Notch1 156,157,158. Moreover, as recently shown in animal models, short peptides such as Aβ38 are not only nontoxic but could even be protective by attenuating the toxicity of Aβ42 *in vivo* 159. Mechanistically, GSMs enhance the processivity of γ-secretase by allowing prolonged residence time of Aβ42 at the enzyme 104, which is probably due to a stabilization of the enzyme-substrate transition state complex 118 through a conformational change in presenilin upon GSM binding 160,161, such that the longer Aβ can be processed to the shorter Aβ. Many highly potent GSMs of various structural classes have been developed 162 and a few of them entered clinical trials and successfully passed phase I. There is hope that proceeding with the development of GSMs could eventually allow safe inhibition of the enzyme without adverse effects 155. Similarly, compounds that would stabilize presenilin in the γ-secretase complex are expected to generally activate the carboxy-peptidase activity in all product lines 118. Certain GSMs such as the bridged aromates fulfill this property since they enhance processivity in both the Aβ40 and Aβ42 product lines leading to increases in both Aβ37 and Aβ38 163. 

Targeting the interaction of C99 with the γ-secretase complex might possibly represent another approach to selectively inhibit Aβ generation. It is conceivable that small compounds might be developable, which could interfere with binding of C99 at the γ-secretase exosites. Unlike the situation with classical GSIs, which bind at or near the catalytic site and are thus expected to provide little if any selectivity of APP over Notch or other substrates, such compounds could interfere with the initial recognition steps at more distant exosites used for substrate binding at stage I. As a consequence, substrates that cannot move to the catalytic site would be released back into the membrane bilayer. Modulating substrate binding at exosites rather than at the catalytic site might thus provide a way to improve substrate selectivity of GSIs. Interestingly, as implied by differences in the Aβ42/40 ratios and that of the corresponding shorter peptides generated from C89 and C83 164, the length of the extracellular domain may be critical for substrate passage and presentation at the active site. Clearly, more mechanistic and structural studies on how γ-secretase recognizes and cleaves its substrates need to be performed but they are clearly warranted and could motivate the development of a new generation of truly substrate-selective γ-secretase inhibitors for clinical testing. It will also be necessary to rule out that GSMs, γ-secretase-stabilizing compounds, or exosite-interaction inhibitors, might, like non-TSA GSIs, cause a potentially unfavorable increase in the levels of the γ-byproducts and of (long) Aβ inside neurons. 

Finally, should ongoing Aβ immunotherapy clinical trials with e.g. the promising aducanumab antibody that successfully removes patient brain amyloid 165 be beneficial in AD, a combination therapy with Aβ42 production modifiers or C99-selective inhibitors to maintain low amyloid levels, thereby preventing and/or shifting the onset of AD, might be an ideal and more cost-effective strategy for disease-modification than an immunotherapy alone.

## Abbreviations

Aβ: amyloid-β
AD: Alzheimer’s disease
AICD: APP intracellular domain
APP: amyloid precursor protein
CTF: C-terminal fragment
EM: electron microscopy
FAD: familial AD
GSI: γ-secretase inhibitor
GSM: γ-secretase modulator
ICD: intracellular domain
NCT: nicastrin
NTF: N-terminal fragment
TMD: transmembrane domain
TSA: transition-stage analogue
wt: wild-type
